# Risky Remedy: Rasburicase-Induced Methemoglobinemia in Tumor Lysis Syndrome Complicated by G6PD Deficiency

**DOI:** 10.7759/cureus.73573

**Published:** 2024-11-13

**Authors:** Supriya Peshin, Sakshi Singal, Nagaishwarya Moka

**Affiliations:** 1 Internal Medicine, Norton Community Hospital, Norton, USA; 2 Medical Oncology, East Tennessee State University, Johnson City, USA; 3 Hematology and Medical Oncology, University of Kentucky College of Medicine, Lexington, USA

**Keywords:** diffuse large b-cell lymphoma, glucose 6 phosphate dehydrogenase (g6pd), methemoglobinemia, rasburicase, tumor lysis syndrome

## Abstract

Tumor lysis syndrome (TLS) is a critical oncologic emergency characterized by metabolic disturbances resulting from rapid cancer cell lysis. Rasburicase, a recombinant urate oxidase, is the primary treatment for hyperuricemia in TLS but poses a risk for methemoglobinemia in patients with glucose 6-phosphate dehydrogenase (G6PD) deficiency. We present the case of a 59-year-old male with diffuse large B-cell lymphoma (DLBCL) who developed spontaneous TLS. Rasburicase was administered, successfully reducing uric acid levels, but the patient subsequently experienced cyanosis, hypoxia, and hemolytic anemia. Methemoglobinemia and G6PD deficiency were confirmed, highlighting the need for early identification of G6PD deficiency before rasburicase use. This case emphasizes the importance of prompt recognition and management of rasburicase-induced methemoglobinemia, especially in high-risk populations.

## Introduction

Tumor lysis syndrome (TLS) is a critical oncologic emergency triggered by the rapid breakdown of cancer cells, typically following treatment. This process releases large amounts of intracellular contents, including nucleic acids, potassium, and phosphates, into the bloodstream, leading to metabolic imbalances. Key disturbances in TLS include hyperuricemia, hyperkalemia, hyperphosphatemia, and secondary hypocalcemia [1]. These can cause serious complications such as acute kidney injury, cardiac arrhythmias, and multi-organ failure.

Rasburicase, a recombinant enzyme that converts uric acid into easily excretable allantoin, is the primary treatment for TLS, effectively reducing uric acid levels [2]. However, in patients with glucose 6-phosphate dehydrogenase (G6PD) deficiency, rasburicase can lead to methemoglobinemia, a rare but severe condition where hemoglobin's ability to carry oxygen is impaired [3, 4]. This reaction necessitates alternative treatments for those at risk. This article was previously presented as a meeting abstract at the Society of Hematology and Oncology's (SOHO) annual meeting on September 6, 2024.

## Case presentation

**Figure 1:**
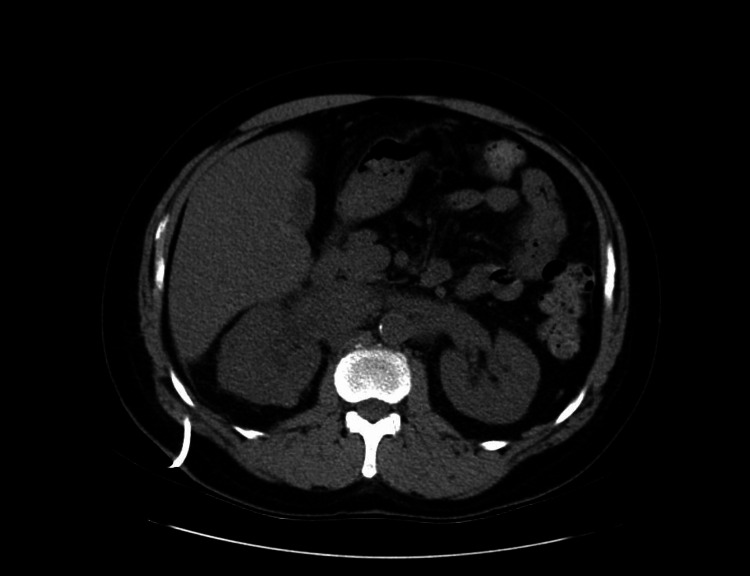
A Computed Tomography (CT) Scan of the Abdomen and Pelvis Revealed a Large Retroperitoneal Mass on the Right Side.

A 59-year-old male presented to the hospital with a medical history of hypertension, non-insulin-dependent diabetes mellitus, gastroesophageal reflux disease (GERD), chronic obstructive pulmonary disease (COPD), not oxygen-dependent, and fibromyalgia that came with worsening right flank pain. Initial laboratory workup revealed an elevated serum creatinine level of 1.5 mg/dL, suggesting mild acute kidney injury. A computed tomography (CT) scan of the abdomen and pelvis identified a large retroperitoneal mass measuring 17 x 16 x 9 cm (Figure 1), involving the right renal hilum and causing severe hydronephrosis. The mass raised concerns for malignancy, and a subsequent biopsy confirmed the diagnosis of diffuse large B-cell lymphoma (DLBCL).

At the time of presentation, the patient also had an elevated serum uric acid level of 10.3 mg/dL, indicating spontaneous TLS. Tumor lysis syndrome is a metabolic emergency commonly associated with aggressive cancers like DLBCL. Given the risk of further complications, immediate treatment was initiated with rasburicase, a recombinant urate oxidase enzyme, to rapidly lower the patient's uric acid levels and prevent renal damage. The initial response to rasburicase treatment was positive, with a reduction in uric acid levels.

However, shortly after receiving rasburicase, the patient developed sudden cyanosis and hypoxia, despite having a normal partial pressure of oxygen (PaO2) on arterial blood gas analysis. This discrepancy between oxygen saturation and PaO2 raised suspicion for methemoglobinemia. Alongside cyanosis, the patient also experienced signs of acute hemolytic anemia, with a significant drop in hemoglobin levels to 5.9 g/dL. Laboratory tests showed markedly elevated lactate dehydrogenase (LDH) at 1,796 U/L, increased indirect bilirubin at 5.0 mg/dL, and decreased haptoglobin (<30 mg/dL), all of which were consistent with hemolysis (Table 1).

**Table 1:** Laboratory Findings in the Case of Rasburicase-Induced Methemoglobinemia

Parameter	Normal Reference Range	Pre-treatment Value	Post-treatment Value
Uric Acid (mg/dL)	(3.5 - 7.2)	10.3	2.0
Hemoglobin (g/dL)	(13.5 - 17.5)	11.2	5.9
Lactate Dehydrogenase (LDH) (U/L)	(140 - 280)	890	1796
Bilirubin (mg/dL)	(0.1 - 1.2)	1.2	5.0
Haptoglobin (mg/dL)	(30 - 200)	60	<30
Methemoglobin (%)	0 - 1.5	-	4.5
Glucose-6-Phosphate Dehydrogenase (U/g)	4.6 - 13.5	-	0.8

Further investigations confirmed methemoglobinemia, with methemoglobin levels measuring 4.5%. Given these findings, the patient underwent testing for G6PD deficiency, which revealed a significantly low G6PD activity level of 0.8 U/g. This confirmed the diagnosis of rasburicase-induced methemoglobinemia and hemolysis in the setting of undiagnosed G6PD deficiency.

The patient received supportive care, including supplemental oxygen and multiple blood transfusions to manage the acute anemia. Rasburicase was immediately discontinued, and the adverse reaction was documented in the patient's medical record to prevent future administration of the drug. The patient’s condition gradually improved with supportive care, and no further complications were reported. On admission, the patient denied fever, chills, chest pain, shortness of breath, back pain, headaches, or myalgias.

This case highlights the importance of early recognition of rasburicase-induced methemoglobinemia in patients with underlying G6PD deficiency, especially in the context of treating TLS. Prompt diagnosis and intervention helped prevent further deterioration in this patient.

## Discussion

Tumor lysis syndrome presents a significant clinical challenge, particularly due to the rapid metabolic changes that occur following the lysis of tumor cells. The metabolic disturbances in TLS, such as hyperuricemia, hyperkalemia, and hyperphosphatemia, require immediate intervention to prevent severe complications, including acute kidney injury and cardiac arrhythmias [1,2]. In this context, rasburicase has emerged as a critical therapeutic agent for managing hyperuricemia associated with TLS, effectively converting uric acid into allantoin, which is more readily excreted by the kidneys [3].

However, the case presented here illustrates the potentially grave consequences of rasburicase administration in patients with undiagnosed G6PD deficiency. G6PD deficiency is an inherited enzymatic disorder that can lead to hemolytic anemia in response to oxidative stress, including exposure to certain medications, foods, and infections [4]. Rasburicase, being a powerful oxidizing agent, can provoke oxidative stress, resulting in methemoglobinemia in such patients [5].

The patient in this case experienced significant adverse effects following the administration of rasburicase, manifesting as cyanosis and hypoxia due to elevated levels of methemoglobin. Methemoglobinemia, characterized by the presence of methemoglobin, leads to impaired oxygen transport because methemoglobin cannot effectively bind oxygen [5]. Symptoms can include cyanosis, fatigue, and, in severe cases, neurological deficits and death if not promptly recognized and treated [6]. This condition was confirmed in our patient with a methemoglobin level of 4.5%, indicating a clinical threshold that warrants immediate intervention [4].

Interestingly, while G6PD testing is straightforward, the urgency associated with treating TLS often delays this important diagnostic step. Therefore, it is essential to maintain a high index of suspicion for G6PD deficiency, especially in patients with a background of African, Mediterranean, or Asian ancestry, who are at higher risk for this condition. Implementing a screening protocol for G6PD deficiency prior to rasburicase administration could significantly reduce the incidence of related complications and improve patient outcomes. To implement an effective G6PD deficiency screening protocol, and establish comprehensive guidelines through collaboration with hematology, oncology, and pharmacy teams, ensuring pre-treatment evaluations for TLS patients include this critical step. Seamlessly integrate laboratory capabilities to enable swift, reliable G6PD testing, supported by standardized procedures for result interpretation and communication. Equip healthcare professionals with targeted training on the importance of screening, rasburicase-related risks, and the management of G6PD-deficient patients, complemented by clear protocols for alternative hyperuricemia therapies. Finally, elevate patient awareness, emphasizing the importance of screening, especially in populations with higher G6PD deficiency prevalence, such as those of African, Middle Eastern, or Mediterranean heritage [4,7].

The management of rasburicase-induced methemoglobinemia typically involves the discontinuation of the offending agent and supportive care, including the administration of supplemental oxygen and blood transfusions if necessary [5]. Methylene blue is also a common treatment for severe methemoglobinemia; however, its use in G6PD-deficient patients is controversial due to the risk of further hemolysis [6,8]. This case emphasizes the importance of tailored therapeutic approaches and vigilant monitoring in high-risk patients, particularly in the setting of oncologic emergencies like TLS.

The prevalence of G6PD deficiency varies greatly across regions and populations, affecting an estimated 400 million people globally. It is most prevalent in areas historically endemic to malaria, as G6PDD provides partial protection against the disease, contributing to its higher frequency in these regions. In Africa, rates range from 5% to 25%, with sub-Saharan populations showing particularly high prevalence. In the Middle East, the prevalence often ranges between 10% and 15%, with some areas recording even higher figures. Mediterranean countries like Italy, Greece, and Cyprus have notable rates between 3% and 30%. In Asia, G6PDD is relatively common, with countries such as Thailand, Malaysia, and parts of China showing rates up to 10% to 20%. Latin American regions generally report lower prevalence, although areas with African or Mediterranean ancestry may have higher rates. In North America and Europe, G6PDD is less frequent but present, mainly among individuals of African, Middle Eastern, or Mediterranean descent, with rates typically around 1% to 2% [9].

Moreover, this incident underscores the necessity for healthcare providers to be well-informed about the potential risks associated with rasburicase, particularly in relation to G6PD deficiency. Continuous education and awareness regarding the interplay of cancer therapies and underlying genetic disorders are crucial in improving patient safety and outcomes in oncologic care.

## Conclusions

This case underscores the importance of early recognition of G6PD deficiency in patients at risk for TLS receiving rasburicase. While rasburicase effectively treats hyperuricemia, its administration in G6PD-deficient patients can lead to severe complications such as methemoglobinemia and hemolytic anemia. Early screening for G6PD deficiency, especially in high-risk populations, should be considered before the initiation of rasburicase. Prompt diagnosis and supportive management are crucial to mitigate the risks of rasburicase-induced methemoglobinemia in these patients.
